# Climate Change and Human Health: Health Impacts of Warming of 1.5 °C and 2 °C

**DOI:** 10.3390/ijerph15061123

**Published:** 2018-05-31

**Authors:** Alice McGushin, Yassen Tcholakov, Shakoor Hajat

**Affiliations:** 1 Faculty of Public Health & Policy, London School of Hygiene & Tropical Medicine, London WC1H 9SH, UK; alice.mcgushin@gmail.com (A.M.); yassen.tcholakov@mail.mcgill.ca (Y.T.); 2 Department of Epidemiology, Biostatistics & Occupational Health McGill University, Montreal, QC H3A 1A2, Canada

In December 2015, a historic agreement was reached at the Paris Climate Conference for the first-ever global deal to reduce greenhouse gas emissions. The legally binding agreement involves “holding the increase in the global average temperature to well below 2 °C above pre-industrial levels and pursuing efforts to limit the temperature increases to 1.5 °C above pre-industrial levels, recognizing that this would significantly reduce the risks and impacts of climate change” [1]. The road to setting these targets has been a long one and originated more in opinion than in evidence, although this has since been rectified and the evidence now exists for many temperature increase scenarios [2].

Whilst recognition of anthropogenic climate change has existed for over half a century, global agreement on what is an acceptable limit to an increase in global average temperature has only been achieved in relatively recent years [3]. To our knowledge, the first written account of a 2 °C target was in 1975 by the economist William Nordhaus in a paper titled “Can We Control Carbon Dioxide?” [4]. The first political acknowledgement of setting a limit to global warming was in 1996, when the European Council of environment ministers stated that “global average temperatures should not exceed 2 degrees above pre-industrial level” [5]. Specific mention of a commitment to this limit, however, did not reach the global stage until the 16th Conference of the Parties (COP16) in Cancun in 2010 [6]. At the same time, Small Island Developing States and Least Developed Countries declared that a 2 °C global increase from pre-industrial temperature was already too much. In the lead-up to COP15 in Copenhagen, 101 countries called for a commitment to a 1.5 °C limit [7]. When negotiations failed to reach an agreement in Copenhagen, the most vulnerable countries renewed their efforts, calling for a 1.5 °C commitment at COP21 in Paris [8].

The Paris Agreement, set to enter force in 2020, includes several mechanisms that allow for global action to achieve the long-term temperature goal, such as mandatory and transparent reporting requirements for countries’ progress on their nationally determined contributions (NDCs) and a global stocktake every five years, the first of which will occur in 2023 [1]. Despite the ambitions of the Paris Agreement, political commitments are still far from reaching the defined goals. Indeed, even with the implementation of all NDCs under the Paris Agreement, there would still be a 3.16 °C increase above pre-industrial levels [9]. Recent estimates suggest a 5% chance of limiting warming to 2 °C and a 1% chance of staying within 1.5 °C [10]. The Talanoa Dialogue aims to address these discrepancies through a facilitative platform to allow inclusive and productive dialogue prior to the completion of the “rulebook” to the Paris Agreement at COP24 in Katowice. The key questions the Talanoa Dialogue is asking are “Where are we?”, “Where do we want to go?”, and “How do we get there?” [11].

Another noteworthy feature of the Paris discussions was that public health was very much enshrined within the agreement. Previously, health was not a major consideration for informing policy on climate change mitigation, but perspective shifted in the Paris Agreement with the realisation that many strategies to reduce carbon emissions, such as improvements in air quality and the switch to more active modes of transport, will also have substantial health co-benefits [12]. Furthermore, health should also be a key consideration in assessments of the differential societal impacts of limiting warming to 1.5 °C as opposed to 2 °C. Given the many additional challenges in achieving the more stringent target, the Intergovernmental Panel on Climate Change (IPCC) will produce a special report, due in September 2018, which will document the additional gains to be made from the 1.5 °C limit. This will include assessments of the range of health impacts associated with climate change. 

The special issue on climate change and human health which details the health impacts of warming of 1.5 °C and 2 °C in the *International Journal of Environmental Research and Public Health* brings together papers highlighting the impacts of climate variability and climate change on population health and health systems. The research examines selected pathways through which climate impacts human health, notably the dangers associated with exposure to hot weather [13,14,15,16] and the role of climate factors in the distribution of vector-borne diseases [17,18]. Authors explore how Shared Socioeconomic Pathways (SSPs)—the new set of future scenarios of societal development designed by the IPCC to guide climate change research—can be extended in health risk assessments [19,20]. Using these SSPs in combination with climate projections, Lee et al. report that achieving a 1.5 °C warming target as opposed to 2 °C would decrease future heat-related mortality in South Korea by about 12%; however, this reduction is likely to be much greater if future demographic changes are also considered [14]. The studies in this collection have been conducted in a range of geographical settings, from Africa to Europe, underscoring the global nature of the problem.

As with the Lee et al. paper, new health risk assessments of climate change would be instructive if they include an explicit comparison of the differential health impacts at the 1.5 °C and 2 °C targets. However, studies which assume that warming remains within 1.5 °C, as opposed to the more likely scenario of breaching this limit before the widespread development of negative emissions technologies can lower temperatures again, will likely underestimate true health burdens. Future risk assessments should also highlight geographical variations in risk as well as consider a wider range of health impacts associated with climate change, including the more indirect processes for which less evidence exists. Articles such as that by Schleussner et al. from 2015, which models the different climate impacts expected at the 1.5 °C and 2 °C targets, provide a good framework upon which comprehensive health risk assessments can be based [21]. That paper reports that the additional 0.5 °C warming would bring about a new climate regime, particularly in tropical regions, which, in turn, would have major impacts on heat-related mortality and morbidity. Reductions in water availability for the Mediterranean is projected to nearly double from 9% to 17%, and the length of regional dry spells would increase from 7% to 11%. Most parts of the world would face substantially lower crop yields at 2 °C, with major implications for global nutritional health; sea-level rise would be reduced by 30% in a 1.5 °C scenario compared to 2 °C.

Given the importance of climate change on human health, healthcare professionals have a crucial role in communicating these impacts to policymakers and to the general public, advocating for strong action on climate change mitigation and adaptation. The health professions have been slow to join the calls for action on climate change, but it appears that this is changing, with many professional organisations having developed policies calling for strengthened action on climate change. Climate change has become one of the top priorities under the World Health Organization (WHO) Director-General, Dr. Tedros Ghebreyesus, with the appointment of Dr. Joy St John as Assistant Director-General on Climate Change and Other Determinants of Health. At the recent 71st World Health Assembly, WHO, alongside the World Meteorological Organization, UN Environment, and the Convention on Biological Diversity, held a technical briefing on health, environment, and climate change, underscoring the importance of intersectoral collaboration on these issues.

The World Medical Association recognizes the importance of keeping the increase in global average temperature to less than 1.5 °C above pre-industrial levels. Furthermore, it urges national governments to “facilitate the active participation of health sector representatives in the creation and implementation of climate change preparedness plans and emergency planning and response on local, national and international levels” [22]. Similarly, the International Federation of Medical Students’ Associations calls governments to “meet an emissions trajectory consistent with a 1.5 degree long term goal”. The health sector is also a great contributor to greenhouse gas emissions and must do what it can to mitigate climate change. Institutions representing more than 10,000 hospitals have signed a “Health Care Call to Action” to make health care systems sustainable [23].

With the WHO describing climate change as *the* defining issue for public health this century, the Paris Agreement is a crucial treaty for this sector. It represents an example of the global cooperation that is required to tackle this global health threat. Researchers and healthcare professionals have a key role to play in characterizing and communicating the health dangers associated with climate change and the gains to be made from achieving emissions targets which will save lives and leave a healthy legacy for current and future generations.
